# A simple tip to improve the accuracy of crossed K-wire placement in the management of displaced paediatric supracondylar fractures of the humerus

**DOI:** 10.1308/003588412X13373405387096c

**Published:** 2012-05

**Authors:** C Leaman, R Kotwal, P Williams

**Affiliations:** Abertawe Bro Morgannwg University Health Board,UK

## BACKGROUND

In 1991 Lindaman *et al* described a fluoroscopic technique to determine the incision site for percutaneous fixation of a slipped capital femoral epiphysis.^1^ Using similar principles, we describe a simple technique to improve the accuracy of crossed K-wire placement in displaced paediatric supracondylar fractures of the humerus. Although not original, our technique is very useful and we believe it should be used more widely when dealing with paediatric elbow fractures as accurate wire placement can be difficult in the presence of swelling and the relative small size of the fracture components in children.

## TECHNIQUE

**Figure 1:**
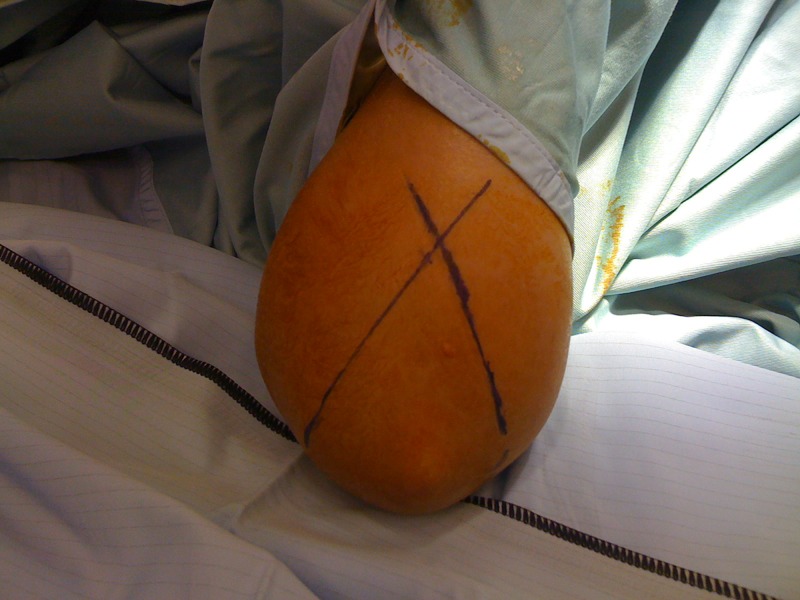
Skin markings for the intended crossed wires

**Figure 2:**
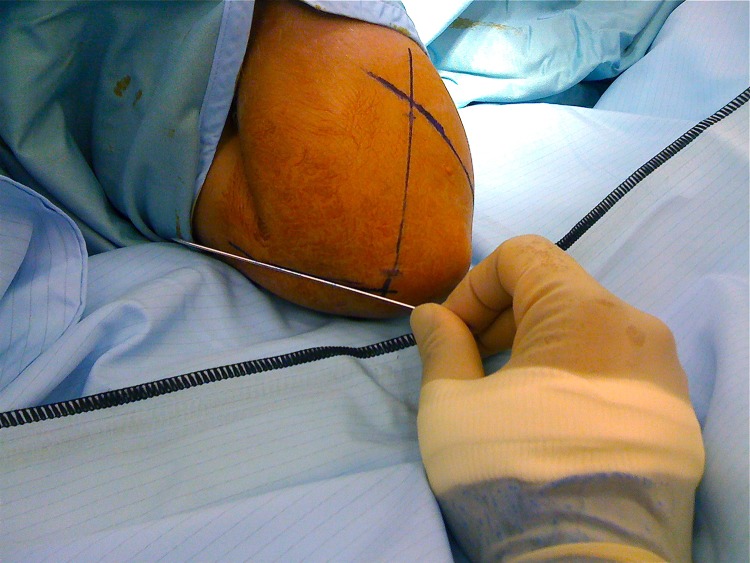
The alignment in lateral view

**Figure 3:**
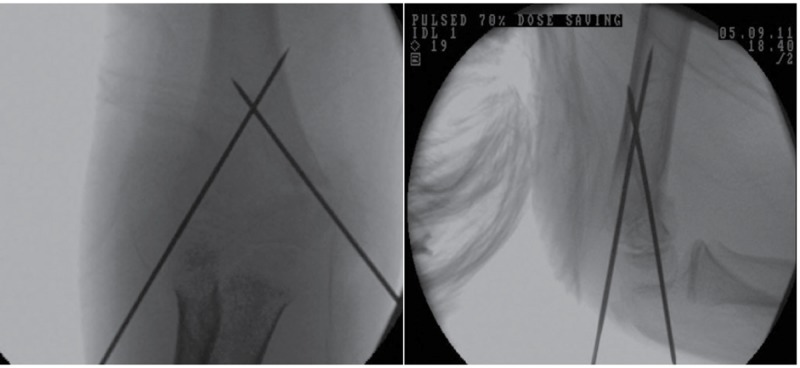
Image intensifier images of placed crossed K-wires: anteroposterior (left) and lateral (right) views

After fracture reduction, the elbow is kept maximally flexed and pronated. K-wires are placed against the skin surface. Alignment is checked with an image intensifier. Three lines are drawn onto the skin. Two of these represent the intended medial and lateral pillar wires (Fig 1). The third line is drawn onto the medial surface (Fig 2) to determine placement in the lateral view. Stab incisions are made in the skin, with mini blunt dissection medially to avoid the ulnar nerve. K-wires (1.6mm) are aligned with the skin markings and driven in under x-ray guidance (Fig 3).

## DISCUSSION

Crossed K-wires are recognised as a stable fixation method.^2^ Accurate wire placement can often be difficult, especially in the presence of significant tissue swelling. Our technique allows the surgeon to visualise a three-dimensional picture of the fracture and the planned fixation before wire placement. This avoids unnecessary soft tissue injury from multiple passes of the wire, reducing the risk of nerve injury and infection.
